# The effect of sarcopenia on erlotinib therapy in patients with metastatic lung adenocarcinoma

**DOI:** 10.17305/bjbms.2022.7147

**Published:** 2022-05-14

**Authors:** Atakan Topcu, Akin Ozturk, Ismail Yurtsever, Mehmet Besiroglu, Ayse Irem Yasin, Haci Mehmet Turk, Mesut Seker

**Affiliations:** 1 Department of Medical Oncology, Faculty of Medicine, Bezmialem Vakif University, Istanbul, Turkey; 2 Department of Medical Oncology, University of Health Sciences Istanbul, Sureyyapasa Chest Diseases and Thoracic Surgery Training and Research Hospital, Istanbul, Turkey; 3 Department of Radiology, Faculty of Medicine, Bezmialem Vakif University, Istanbul, Turkey

**Keywords:** Lung cancer, sarcopenia, erlotinib, adenocarcinoma, prognosis

## Abstract

Erlotinib, a tyrosine kinase inhibitor, has been shown to improve the survival of patients with epidermal growth factor receptor (EGFR)-mutated non-small cell lung cancer. Sarcopenia is a status with increasing importance in lung cancer, and it may predict a poor prognosis. We aimed to evaluate the impact of sarcopenia on erlotinib therapy and prognosis in patients with EGFR-mutated (exon 19 or 21 L858R) metastatic lung adenocarcinoma. Sarcopenia was defined as skeletal muscle index ≤39 cm^2^/m^2^ for women and ≤55 cm^2^/m^2^ for men. The patient characteristics, inflammation parameters, clinical and survival outcomes of the erlotinib therapy were examined according to sarcopenia status. We also analyzed the erlotinib treatment-related toxicity. Seventy-two patients were included in our retrospective study, and the mean age of the patients was 63.7 years. A total of 39 (54.2%) patients were diagnosed with sarcopenia. Patients with sarcopenia had a poor prognosis and had a shorter median progression-free survival (PFS) than patients without sarcopenia (10.5 months vs. 21.8 months, *p* = 0.002). Sarcopenia (HR 2.08) and C-reactive protein > 6.5 mg/L (HR 2.57) were determined as independent poor prognostic factors for PFS of erlotinib therapy. Treatment-related toxicity occurred in 34.7% of patients treated with erlotinib, and sarcopenia did not significantly affect treatment-related toxicity. We also found that sarcopenia significantly affected the response to erlotinib. The expected survival outcomes may be low when erlotinib therapy is used in patients with sarcopenia and metastatic lung adenocarcinoma. This study showed that survival and clinical outcomes could be better predicted by detecting sarcopenia in patients with lung cancer using erlotinib.

## INTRODUCTION

Lung cancer is one of the most common cancers worldwide. It is the leading cause of death in both sexes [1]. Non-small cell lung cancer (NSCLC) represents the majority of all lung cancers. Adenocarcinoma is the most common type of lung cancer and accounts for half of all lung cancer cases [2]. Epidermal growth factor receptor (EGFR) mutations in EGFR tyrosine kinase have been detected in approximately 15% of NSCLC. These genetic alterations may lead to the activation of EGFR signaling, thus stimulating cell proliferation, migration, and survival [3]. In advanced NSCLC, the presence of EGFR mutations, exon 19 deletions or exon 21 L858R mutations, strongly predicts responsiveness to EGFR tyrosine kinase inhibitors (TKIs), such as erlotinib, resulting to a more favorable prognosis. Erlotinib has been shown to significantly improve the survival of patients with EGFR-mutated NSCLC [4,5]. The dose of erlotinib is 150 mg once daily until the unacceptable toxicity or disease progression. Treatment-related toxicities which can occur during erlotinib therapy are dermatologic, gastrointestinal, ocular, neuromuscular, skeletal, and pulmonary toxicities [6,7].

Sarcopenia is defined by a triad of loss of skeletal muscle mass, muscle strength, and physical performance [8]. However, loss of skeletal muscle mass as measured by computed tomography (CT) is still considered a valid method for defining sarcopenia in patients with cancer. Thus, sarcopenia can be simply evaluated with routine CT scans in clinical practice in patients with cancer [9-11]. Factors contributing to the development and progression of sarcopenia include cachexia, uncontrolled weight loss, physical inactivity, aging, use of drugs, and body absorption dysfunction. Most patients with lung cancer can be affected by cancer cachexia at the time of diagnosis or during treatment. Cancer cachexia is associated with systemic inflammation and sarcopenia, both of which are independently associated with poor prognosis of patients with cancer [12,13]. The progressive loss of muscle mass and consequent loss of strength result in decreased quality of life, physical disability, increased toxicity of the therapeutic drug, and increased mortality rates. Sarcopenia may occur in approximately 50% of all lung cancer cases and predicts a poor prognosis [11,14-16]. However, this has not yet been demonstrated in patients with lung cancer receiving erlotinib therapy.

Our study aimed to evaluate the impact of sarcopenia on erlotinib therapy and prognosis in patients with metastatic lung adenocarcinoma harboring EGFR mutations. We also analyzed the erlotinib treatment-related toxicity.

## MATERIALS AND METHODS

### Study design and sample

This retrospective study used the files of patients diagnosed with histologically confirmed lung adenocarcinoma harboring EGFR mutations in exon 19 or exon 21 L858R in two oncology centers between years 2013 and 2020. Patients who received first-line erlotinib or first-line platinum-based chemotherapy followed by second-line erlotinib during the metastatic period were included in our study. Patients who did not receive erlotinib, who received erlotinib after the second-line, those with other EGFR mutations, those who did not have a CT imaging within one month before starting erlotinib therapy, those who could not be evaluated within eight weeks of initiation of erlotinib, those with Eastern Cooperative Oncology Group Performance Status (ECOG PS) >3, those under 18 years of age, those with uncontrolled comorbid diseases, those with a history of intestinal malabsorption, those with clinical manifestations of acute infectious disease, and those with other histological subtypes such as squamous cell carcinoma were excluded.

### Patient evaluation

General clinical characteristics of patients were noted retrospectively. Patients were started with erlotinib dose of 150 mg once daily, as first- or second-line treatment in the metastatic setting by their clinician until disease progression, intolerable toxicity, or death. Routine monitoring parameters of erlotinib were assessed and noted by the clinicians in both centers. Treatment-related toxicity could lead to dose reduction, treatment delay, or drug withdrawal. Inflammation parameters were measured prior to initiating erlotinib treatment. Disease evaluations were performed with standard CT and treatment response was analyzed using the Response Evaluation Criteria in Solid Tumors version 1.1. Objective response rate (ORR) was defined as the sum of a complete or partial erlotinib response. Disease control rate (DCR) was defined as the sum of complete response, partial response, or stable disease. Progressive disease (PD) was diagnosed based on the appearance of new lesions and an increase of ≥20%. In size of the preexisting lesion, progression-free survival (PFS) was the time from the date of initiation of erlotinib until first PD or death. Overall survival (OS) was the time from the date of initiation of erlotinib until the last date on which the patient was known to be alive, or death.

### Imaging evaluation

The CT imaging for sarcopenia evaluation was performed within one month before starting erlotinib. The CTs were examined by an experienced radiologist (IY). The radiologist used semiautomatic software (Syngo. via, Siemens, Germany) for total muscle area (TMA) evaluation and was blind to other data of subjects at the time of scans evaluation. The third lumbar vertebra (L3) was preferred as the standard landmark because it corresponds best with whole-body muscle mass [17]. For the evaluation of sarcopenia, skeletal muscle index (SMI; cm^2^/m^2^) was calculated. SMI represents the total cross-sectional skeletal muscle area (TMA; cm^2^) at the level of L3. SMI was computed as TMA at L3/(height × height). Sarcopenia was defined by international consensus as an SMI of ≤ 39 cm^2^/m^2^ for women and ≤ 55 cm^2^/m^2^ for men [18]. The Clinical Research Ethics Committee of Bezmialem Vakif University reviewed and approved this study with the decision number: 21/390.

### Statistical analysis

**FIGURE 1:**
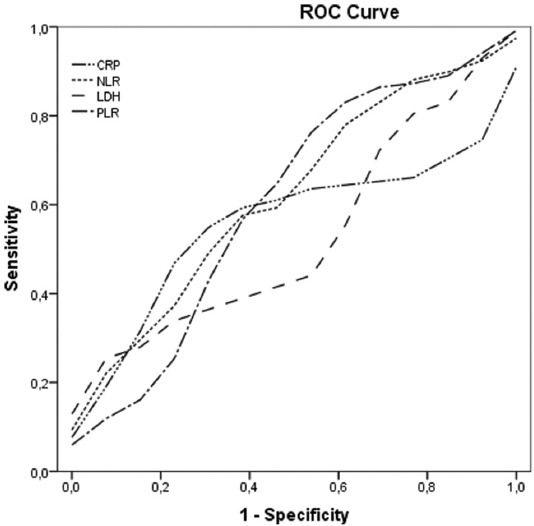
Receiver operating characteristic (ROC) curve for CRP (AUC =0.56), NLR (AUC = 0.62), LDH (AUC = 0.53), and PLR (AUC = 0.61). CRP: C-reactive protein; NLR: neutrophil-to-lymphocyte ratio; LDH: lactate dehydrogenase; PLR: platelet-to-lymphocyte ratio.

**FIGURE 2:**
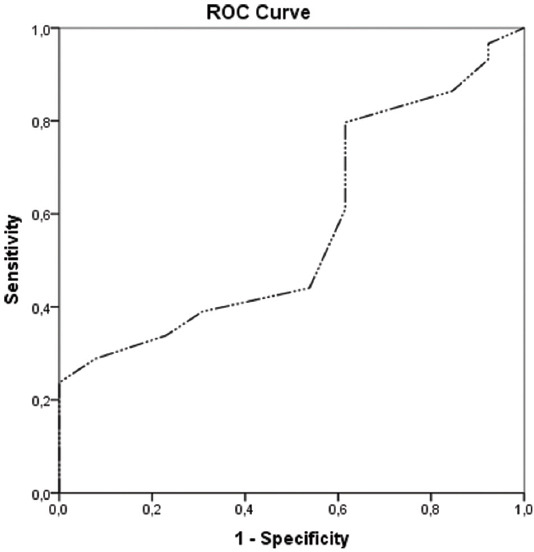
Receiver operating characteristic (ROC) curve for albumin (AUC =0.57).

Statistical analysis was conducted using IBM^®^ SPSS^Ò^ Statistics Version 24.0 software package. Qualitative variables were described by frequencies and percentages, continuous and ordinal variables by mean and standard deviation, or median and range. The normal distribution range was determined by the Kolmogorov–Smirnov test. Qualitative variables were compared using the Pearson χ^2^ test. The characteristics of patients were evaluated with descriptive analysis. The median cutoff values for C-reactive protein (CRP), lactate dehydrogenase (LDH), platelet-to-lymphocyte ratio (PLR), and neutrophil-to-lymphocyte ratio (NLR) were determined by receiver operating characteristic (ROC) curve analysis. The median cutoff values for C-reactive protein (CRP), lactate dehydrogenase (LDH), platelet-to-lymphocyte ratio (PLR), and neutrophil-to-lymphocyte ratio (NLR) were determined by receiver operating characteristic (ROC) curve analysis (Figures 1 and 2). Survival analysis was performed using the log-rank test and Kaplan–Meier survival curves. Univariate and multivariate Cox proportional hazard models were used to identify predictors of PFS. Hazard ratios (HRs) with 95% confidence intervals (CIs) were applied to quantify the indexes estimating the survival. Statistical significance was accepted at *p* < 0.05.

## RESULTS

Seventy-two patients met the eligibility criteria and were included in this study. Most patients were female (65.3%) and never smokers (66.7%). The mean age was 63.7 ± 11.3 years, and 57 (79.2%) patients had an ECOG PS 0-1. Exon 19 deletions were detected in 62.5% of patients and exon 21 L858R point mutations in 37.5% of patients. All patients had adenocarcinoma histological subtype. Of the 72 patients, 50 (72.2%) had metastatic disease at diagnosis, and 45 (62.5%) used erlotinib as first-line therapy. 27.8% of patients had brain metastases. Patients were divided into two groups, based on their SMI. A total of 39 (54.2%) patients were defined with sarcopenia. Sarcopenia was significantly more common in males (*p* = 0.001), in patients with ECOG PS 2-3 (*p* = 0.024), in non-obese patients (p = 0.001), and in patients with cranial metastasis (*p* = 0.006). Comparison of baseline characteristics between sarcopenic and non-sarcopenic group is shown in Table 1.

**TABLE 1:**
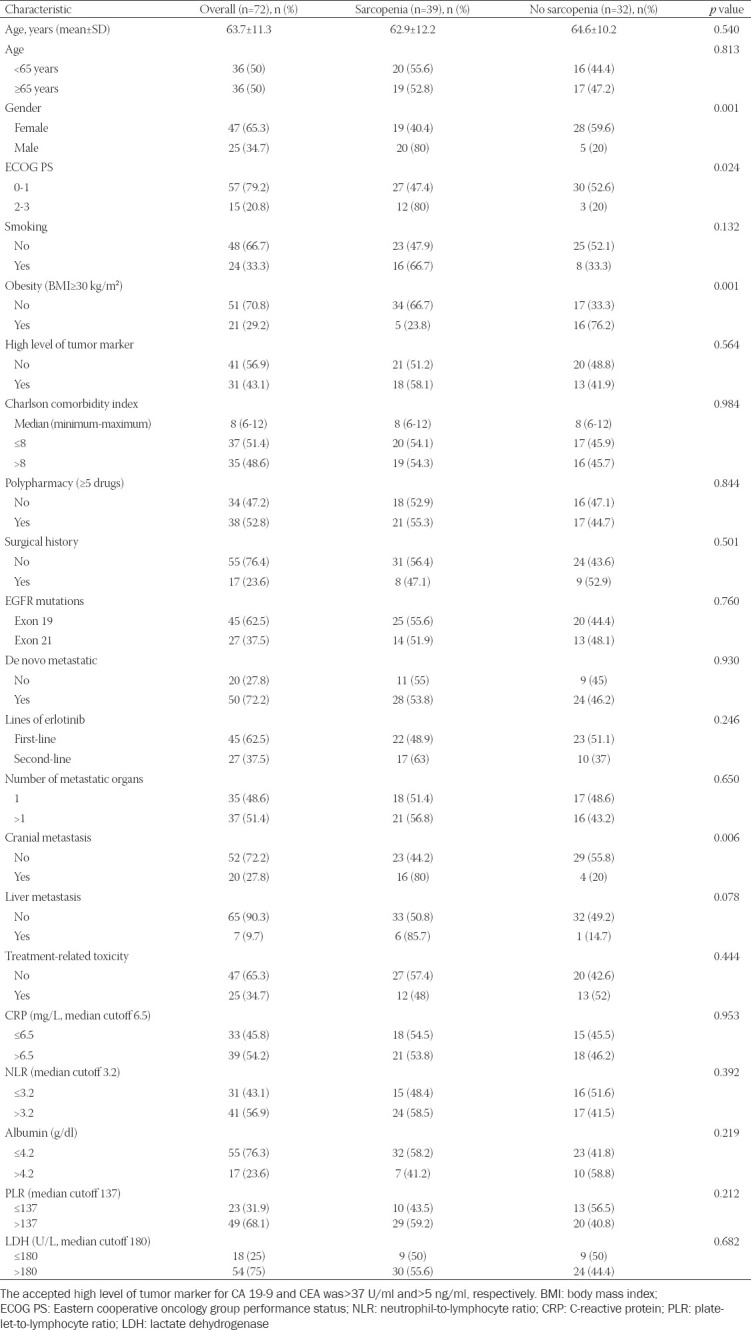
Baseline characteristics of patients

Median L3 SMI was 42.2 cm^2^/m^2 (^24.6-70.3 cm^2^/m^2^), average body mass index (BMI) was 28.2 ± 5.4 kg/m^2^, and 29.2% of the patients were obese. Comparison of body composition variables between sarcopenic and non-sarcopenic group is shown in Table 2.

**TABLE 2:**
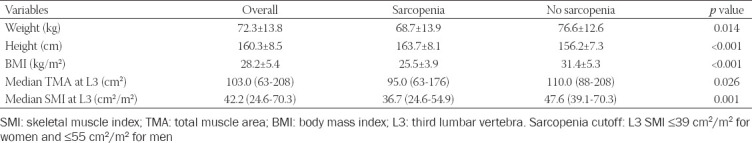
Comparison of body composition variables between sarcopenic and non-sarcopenic patients.

Treatment-related toxicity occurred in 25 (34.7%) patients. It was detected in 12 (30.8%) of those with sarcopenia, and 13 (39.4%) of those without sarcopenia, and no important difference in treatment-related toxicity status between two groups was observed (p = 0.444). The most frequently reported adverse events associated with erlotinib by their clinicians during routine clinical practice were skin rash, fatigue, nausea, diarrhea, and increased serum creatinine, ordered from most to least common. While the dose of erlotinib was reduced in 21 of 25 patients, it was stopped in 4 patients. In addition, the relationship between treatment-related toxicity and the PFS of erlotinib was analyzed. The median PFS was 15.8 months (8.4-23.2) in treatment-related toxicity group, while the median PFS was 11.0 months (7.6-14.4) in non-treatment-related toxicity group, which were not significantly different (*p* = 0.074).

**FIGURE 3:**
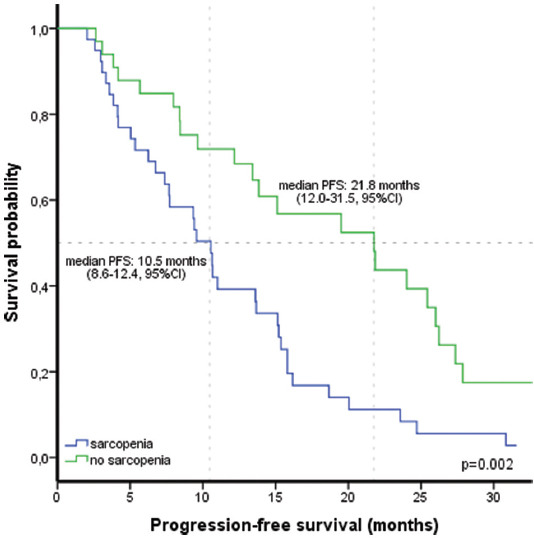
Kaplan-Meier curves of progression-free survival in patients with and without sarcopenia. PFS: progression-free survival.

**FIGURE 4:**
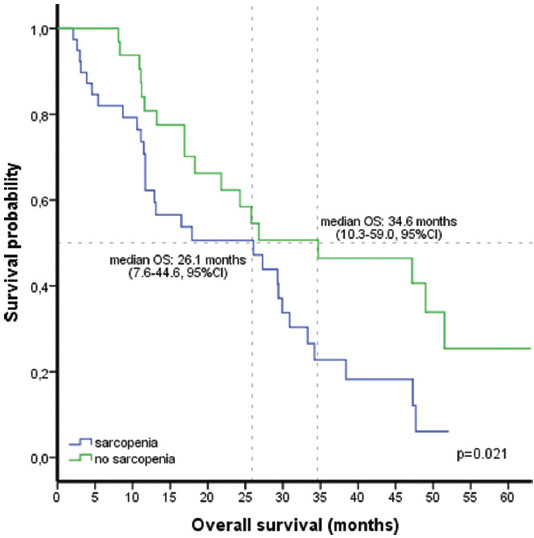
Kaplan-Meier curves of overall survival in patients with and without sarcopenia. OS: overall survival.

With a median follow-up time of 17.4 months (2.0-63.9), in 59 patients (82%) occurred disease progression. In the whole cohort of patients, the median PFS and OS were 13.6 months (9.0-18.2, 95% CI) and 26.7 months (95% CI 19.2-34.2), respectively. The rate of patients who reached median PFS was 78% at 6 months, 54% at 1 year, and 22% at 2 years. The rate of patients who reached median OS was 71% at 1 year, 56% at 2 years, and 34% at 3 years. Patients were divided into two main groups based on their sarcopenia status. Disease progression was observed in 36 of 39 patients in the sarcopenic group and in 23 of 33 patients in the non-sarcopenic group. Patients with sarcopenia had a shorter median PFS than patients without sarcopenia (10.5 months (95% CI 8.6-12.4) vs. 21.8 months (95% CI 12.0-31.5), *p* = 0.002; Figure 3). On the other hand, patients with sarcopenia had a shorter median OS than patients without sarcopenia (26.1 months (95% CI 7.6-44.6) vs. 34.6 months (95% CI 10.3-59.0), *p* = 0.021; Figure 4). In addition, the rates of sarcopenia were higher in males. In the subgroup analysis of gender for PFS between sarcopenic and non-sarcopenic groups, similar results were obtained in females (11.0 months vs. 24.0 months, *p* = 0.009). However, no significant difference was found in men (9.3 months vs. 5.7 months, *p* = 0.461).

The univariate analysis of the clinical and demographic characteristics for PFS identified male gender (HR 3.19), ECOG PS 2-3 (HR 2.04), smoking (HR 3.04), sarcopenia (HR 2.35), cranial metastasis (HR 2.20), liver metastasis (HR 2.92), and CRP > 6.5 mg/L (HR 2.46) as poor prognostic factors. As a result of the multivariate analysis for PFS, sarcopenia (HR 2.08) and CRP > 6.5 mg/L (HR 2.57) were determined as an independent poor prognostic factors. The results of univariate and multivariate analyses of factors related to PFS of erlotinib are detailed in Table 3.

**TABLE 3:**
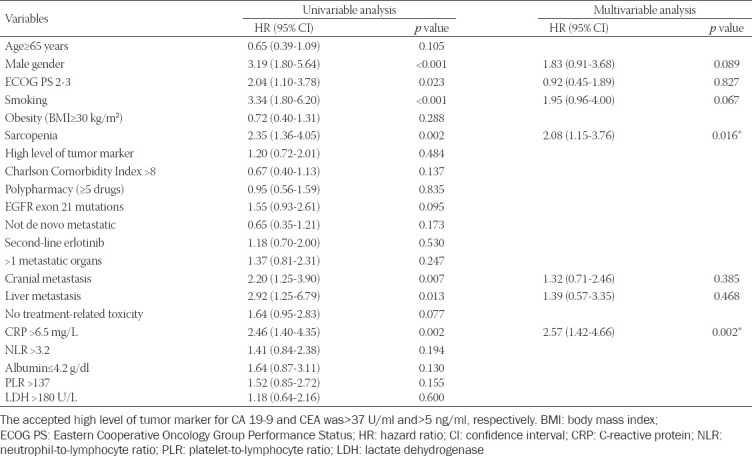
Results of univariable and multivariable analyses of progression-free survival.

**FIGURE 5:**
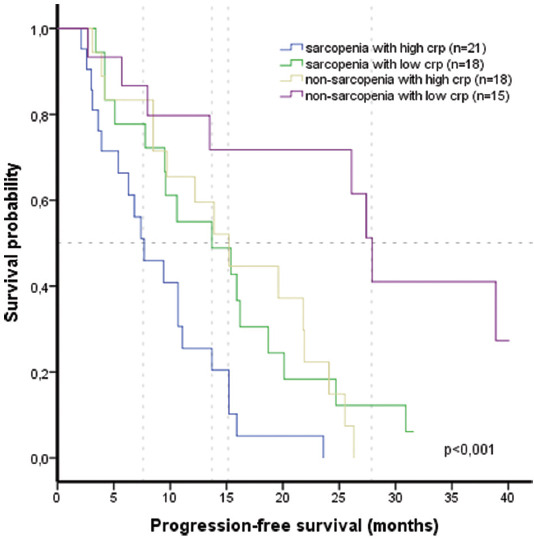
Kaplan–Meier curves of progression-free survival by the risk groups. *Median PFS by the risk groups: sarcopenia with high CRP level group: 7.7 months (95% CI 4.0–11.4); sarcopenia with low CRP level group: 13.7 months (95% CI 4.4–23.0); non-sarcopenia with high CRP level group 15.1 months (95% CI 10.2–20.0); non-sarcopenia with low CRP level group 27.9 months (95% CI 25.2–30.6). PFS: progression-free survival; CRP: C-reactive protein.

The participants were classified into four groups based on their sarcopenia and CRP level status, both of which were determined to be poor prognostic factors for PFS, and median PFS of these groups were compared (Figure 5). The median PFS for sarcopenia with high CRP level group was 7.7 months (95% CI 4.0-11.4), sarcopenia with low CRP level group was 13.7 months (95% CI 4.4-23.0), non-sarcopenia with high CRP level group was 15.1 months (95% CI 10.2-20.0), and non-sarcopenia with low CRP level group was 27.9 months (95% CI 25.2-30.6). There was an important difference in median PFS between these groups (*p* < 0.001).

In addition, the effects of sarcopenia and CRP level on erlotinib response were analyzed. Patients with sarcopenia had a lower ORR than patients without sarcopenia (38.5% [*n* = 15] vs. 63.6% [*n* = 21], *p* = 0.033). Similarly, patients with sarcopenia had a lower DCR than patients without sarcopenia (69.2% [*n* = 27] vs. 93.9% [*n* = 31], *p* = 0.008). Furthermore, patients with sarcopenia had a higher rate of PD than patients without sarcopenia (30.8% [*n* = 12] vs. 6.1% [*n* = 2], *p* = 0.008). In addition, no significant differences in the ORR, DCR, and PD were observed between the groups with high or low CRP levels (43.6% vs. 57.6%, *p* = 0.237; 76.9% vs. 84.8%, *p* = 0.397; and 23.1% vs. 15.2%, *p* = 0.397, respectively).

Patients with disease progression were divided into two subgroups: sarcopenic and non-sarcopenic. Non-sarcopenic patients had a significantly higher chance of receiving the next-line therapy after erlotinib than sarcopenic patients (65.2% vs. 48.3%, *p* = 0.049). In addition, there was no significant difference in receiving osimertinib treatment after erlotinib between the sarcopenic and non-sarcopenic group (22.2% vs. 39.1%, *p* = 0.162).

## DISCUSSION

Sarcopenia is the main feature of cancer cachexia, which is common in patients with lung cancer and can predict a poor prognosis [14]. Cachexia involves various mediators derived from the cancer cells, which are associated with systemic inflammation. It is also noted that some pathophysiological mechanisms may be related to the loss of muscle mass in patients with cancer. These causes are impaired mitochondrial function, differentiation of insulin-mediated glucose metabolism, the increased metabolic activity of more aggressive tumor biology, the pharmacokinetics of the drugs, decreased myokine production, poor nutritional status, decreased quality of life, physical disability, and increased mortality rate. This may clarify why sarcopenia is a poor prognostic factor [19]. The growing importance of the association between sarcopenia and lung cancer outcomes makes it a substantial target for future approaches.

This study presented a considerable real-life data on the effect of sarcopenia on erlotinib therapy. We found that sarcopenia had a significant impact on the survival of patients with lung adenocarcinoma treated with erlotinib. In contrast, sarcopenia did not significantly affect treatment-related toxicity in our research. In this study, sarcopenia and high CRP levels (systemic inflammation) were detected as independent risk factors on the PFS of erlotinib therapy. Furthermore, treatment-related toxicity status did not statistically affect the PFS. Besides, no statistical significance was found for other baseline parameters.

The importance of sarcopenia on the prognosis of lung cancer patients treated with TKI is increasing. Nie et al. showed that PFS of afatinib in patients with lung cancer was similar in those with or without sarcopenia. They found that sarcopenia was a risk factor for treatment-related toxicity [9]. In a retrospective study that evaluated gefitinib outcomes in patients with EGFR-mutated NSCLC according to their sarcopenia status, they found that sarcopenia did not affect PFS and treatment-related toxicity. However, sarcopenia was a poor risk factor for OS in this study [11]. In a Canadian study, although there was a numerical difference, no significant association was found between sarcopenia and treatment-related toxicity, PFS, and OS in lung cancer patients using afatinib [16]. In a Japanese study, sarcopenia was found not to affect PFS in patients using TKI. Low BMI was shown as the poor prognostic factor for the PFS [20]. However, in this study, sarcopenia was defined using different techniques such as psoas muscle index, visceral subcutaneous adipose tissue, and intramuscular adipose tissue. This may have led to different results. There may also be racial and geographical differences. In contrast, Ono et al. presented no relationship between BMI and PFS in NSCLC patients treated with osimertinib [21]. In our research, 54.6% of patients were diagnosed with sarcopenia, and we found that patients with sarcopenia had a shorter median PFS than patients without sarcopenia (10.5 months vs. 21.8 months, *p* = 0.002). Furthermore, patients with sarcopenia had a shorter median OS than patients without sarcopenia (26.1 months vs. 34.6 months, *p* = 0.021). Our study determined that sarcopenia was an independent poor prognostic factor for PFS of erlotinib.

Erlotinib provides a survival advantage in patients with EGFR-mutated NSCLC. In recent studies, OS and PFS of erlotinib have been approximately 23 months and 13 months, respectively [22,23]. Similarly, in our study, the median OS and PFS in all patients were 26.7 months and 13.6 months, respectively. In general, sarcopenia was detected in approximately half of the patients with lung cancer and might be a significant predictor for poor response to the treatment and worse survival rates [9,11,14].

In the present study, the patients with sarcopenia had a poor prognosis. Moreover, sarcopenic patients had a lower ORR and clinical response than non-sarcopenic patients. There was no difference in treatment-related toxicity between the sarcopenic and non-sarcopenic group. We think that the negative effects of sarcopenia on the erlotinib response may have contributed to the worse prognosis in our study. Similar results have been previously reported in studies on immunotherapy in lung cancer [10,24,25]. However, there is no clear information on erlotinib yet. Nonetheless, this result may not surprise given that sarcopenic patients have a significant risk factor for poor outcomes. Our study provided a perspective on this issue, and more extensive studies should also confirm it. In addition, our study found that non-sarcopenic patients had a significantly higher chance of receiving the next-line therapy after erlotinib than sarcopenic patients (*p* = 0.049). There was also no significant difference in receiving osimertinib treatment after erlotinib between the sarcopenic and non-sarcopenic group (*p* = 0.162). We believe that, due to the poor prognosis of sarcopenia, sarcopenic patients may have inadequate access to the next-line treatments, which may negatively contribute to their survival.

The relationship between sarcopenia and treatment-related toxicity is unclear in patients with lung cancer [13,14,26,27]. The studies have found similar treatment-related toxicity status in patients with sarcopenic and non-sarcopenic lung cancer treated with gefitinib and afatinib [11,16]. In contrast, Nie et al. presented that treatment-related toxicity occurred more frequently in patients with sarcopenic lung cancer using afatinib [9]. The small number of patients and race/ethnicity differences among the patient groups may have led to different results. The rate of treatment-related toxicity was reported in about 50% of patients on TKI treatment in these studies. In our study, treatment-related toxicity occurred in 34.7% of patients treated with erlotinib, in 30.8% of patients with sarcopenia, and in 39.4% of patients without sarcopenia. However, no relationship was found between treatment-related toxicity status and sarcopenia (*p* = 0.444). The previous studies have found that PFS was not different in patients with or without treatment-related toxicity of afatinib in lung cancer [9,16,28]. In our study, treatment-related toxicity did not significantly affect the PFS of erlotinib (*p* = 0.074). Thus, we suggest that in sarcopenic patients, tolerable dose administration with close monitoring may be possible.

Sarcopenia is associated with cancer-related inflammation, and both are associated with worse survival in patients with cancer [12]. Thus, including both sarcopenia and inflammatory biomarkers may help to better explain the prognosis of these patients. In addition, the systemic inflammatory response (SIR) plays a crucial role in cancer. Serum CRP level is a helpful sign of SIR and is generally used as a systemic marker of inflammation [29]. Elevated serum CRP level may be a sign of a high tumor burden or catabolic effects on the metabolism [30]. Several studies have revealed that high CRP level was an independent poor prognostic factor for the survival of patients with metastatic lung cancer receiving chemotherapy [31,32]. Moreover, systemic inflammation may be closely associated with the progression of sarcopenia [33]. In a meta-analysis, sarcopenia was associated only with high CRP level [34]. In the present study, there was no difference between the sarcopenic and non-sarcopenic group in terms of the inflammatory status. This was also demonstrated in other studies [13,35]. In addition, it is known that cancer cachexia and sarcopenia can be present without overt systemic inflammation [18]. In a Korean study, sarcopenia and high NLR, an inflammation marker, were found as independent risk factors for shorter PFS in patients with small cell lung cancer receiving chemotherapy. This study suggests that these patients do not tolerate standard treatment, and intensive supportive care may be needed [13]. In a Croatian study, it was determined that high CRP level negatively affected PFS in patients with NSCLC receiving chemotherapy. In this study, sarcopenia was determined in 47% of the patients, but sarcopenia did not affect the PFS and treatment-related toxicity [27]. Sarcopenia and PLR were found to have a combined effect on the survival of patients with advanced cancer using immunotherapy. However, number of the patients was relatively low, and PLR was the only inflammation marker evaluated in this study [12]. In an Austrian study conducted in patients with NSCLC, it was found that high CRP level (≥ 10 mg/dl) significantly affected the PFS of gefitinib. High CRP level was detected in 68% of the patients [36]. Similarly, in patients with NSCLC, Masago et al. found that the PFS of gefitinib was statistically lower in high CRP level (cut-off analysis, 10 mg/dl) group. High CRP level was present in 32.9% of the patients [37]. In a study in patients with NSCLC, high CRP level was determined to be an independent prognostic factor for the PFS of erlotinib. However, when the subgroups were analyzed, high CRP level did not affect the PFS in EGFR-mutated patients. In addition, wild-type EGFR and ECOG PS 2-3 adversely affected the PFS of erlotinib. However, while there were 37 patients with EGFR mutation, the effect of poor performance scores in this subgroup was unknown [29]. In our study, both high CRP level and sarcopenia were detected in 54.2% of patients. We found that high CRP level and sarcopenia were independent poor prognostic factors for PFS in patients with lung cancer using erlotinib. In our research, the presence of sarcopenia (HR 2.08, 95% CI 1.15-3.76) and high CRP level (HR 2.57, 95% CI 1.42-4.66) were associated with the worst PFS (median 7.7 months), whereas the absence of these two factors was associated with the best PFS (median 27.9 months). However, the NLR level was not significantly associated with sarcopenia in our study. In the previous report, LDH was used to evaluate inflammation. There was no difference in LDH values between sarcopenic and non-sarcopenic groups. LDH was not a significant prognostic factor for PFS in this study. Similar results were obtained in our study [38]. In addition, our study presented that there had been no difference in PFS between EGFR exon 19 and 21 L858R mutations in lung adenocarcinoma. In other studies, it is also stated that there may not be a significant difference in PFS between EGFR exon 19 and 21 mutations [4,5].

The preferred method for evaluating sarcopenia is the calculation of SMI using CTs. In our study, psoas density (muscle quality) was not included. For this purpose, all cases should be imaged with the same device and protocol. In addition, skeletal deformity disorders in patients can result in muscle atrophy and hypertrophy in the contralateral muscle, creating false impressions during evaluation. Moreover, degenerative osteophytes and hyperostosis may create scattering artifacts and impair the density optimization [39].

In the study evaluating the effect of sarcopenia in patients with lung cancer treated with gefitinib, TMA (median 103.0 cm^2^ vs. 106.6 cm^2^) and SMI (42.2 cm^2^/m^2^ vs. 40.2 cm^2^/m^2^) values, the components of sarcopenia diagnosis, were similar to those in our study [11]. The mean age of the patients in this study was 66, and 82% were female. Sarcopenia was found in 60% of the patients in the abovementioned study. In our study, sarcopenia was found in 54.6% of the patients. The mean age was 63.7 years, and 65.3% were women. In addition, the mean weight, BMI, median TMA, and SMI of patients with sarcopenia were lower than of patients without sarcopenia, whereas the mean height was higher.

The limitations of our research are its retrospective study nature and small sample size. We think more precise recommendations can be given if the number of patients is increased in future studies. Since our study involves two centers, the CT machines may not be the same for all patients, reducing the data standardization. In our study, we defined sarcopenia using only muscle quantity. As a result, muscle strength and physical performance were not considered when defining sarcopenia. However, the preferred method for defining sarcopenia is still the evaluation of muscle mass loss with CTs in patients with cancer. Nevertheless, assessing sarcopenia along with muscle strength may allow us to better analyze this area in future studies. In addition, the detailed adverse events and toxicities were not included, although they were mentioned generally in this study. However, further research is needed to understand the relationship between systemic inflammation and sarcopenia, which is of particular significance in lung cancer treatment.

## CONCLUSION

We examined the effect of sarcopenia on erlotinib therapy and prognosis in patients with metastatic lung adenocarcinoma. Patients with sarcopenia had worse prognosis in our study. Sarcopenia and high CRP level were independently associated with PFS of erlotinib as poor prognostic factors. However, sarcopenia did not significantly affect treatment-related toxicity. In addition, we found that sarcopenia significantly affected the response to erlotinib. The expected survival outcomes may be low when erlotinib therapy is used in patients with sarcopenia. This study showed that survival and clinical outcomes could be better predicted by detecting sarcopenia in patients with lung cancer using erlotinib. Furthermore, identifying sarcopenic patients before starting erlotinib and providing treatment for sarcopenia might increase the survival benefit of the patients. Thus, comprehensive prospective studies with more patients must be conducted on this subject.
